# A Systematic Review of Clinical Practice Guidelines for Castration-Resistant Prostate Cancer

**Published:** 2018

**Authors:** Mohamad Javad Foroughi Moghadam, Saeed Taheri, Farzad Peiravian

**Affiliations:** *Department of Pharmacoeconomics and Pharmaceutical Management, School of Pharmacy, Shahid Beheshti University of Medical Sciences, Tehran, Iran.*

**Keywords:** Treatment Guideline, Hormone-Refractory Prostate Cancer, Health-Related Quality of Life, Cost, Enzalutamide, Abiraterone Acetate, Cabazitaxel

## Abstract

Cancer constitutes a huge burden on societies in countries with any level of economic development. Prostate cancer is the first most diagnosed cancer of men in developed countries and the forth one in developing countries in terms of incidence rate. It is also the third incident cancer of men in Iran along with a prevalence of about 10,000 cases. Castration-resistant prostate cancer (CRPC) is a severe stage of the disease with a number of newly discovered treatment options. These therapeutic alternatives including abiraterone acetate, enzalutamide, cabazitaxel, immunotherapy with sipuleucel-T, radiopharmaceuticals and bone-targeted therapies (zoledronic acid, denosumab) along with docetaxel have made the decision making process complex and challenging for clinicians. In addition to the challenges of selecting the best-fit treatment, high costs of new pharmaceuticals and technologies necessitates the health policy-makers to develop practice guidelines in adaptation with local resources and limitations. The aim of this paper is to review the clinical guidelines for the management of CRPC. For better comprehension of guideline recommendations, the main clinical trials on new treatments were also identified. The efficacy and safety outcomes including but not limited to overall survival, progression free survival, quality of life and adverse effects were summarized. The guidelines of American Urological Association (AUA), National Comprehensive Cancer Network (NCCN), European Association of Urology (EUA), Spanish Oncology Genitourinary Group (SOGG), Asian Oncology Summit, Saudi Oncology Society-Saudi Urology Association combined guideline, National Institute for Health and Care Excellence (NICE) and Canadian Urological Association-Canadian Urologic Oncology Group (CUA-CUOG) were covered in this paper.

## Introduction

*Epidemiology*

Cancer constitutes a huge burden on societies in countries with any level of economic development. The increasing trend in the occurrence and prevalence of cancer is a result of complex reasons including population growth, aging, lower physical activity, obesity, smoking and so many other underlying conditions of urbanization and economic development (1). Prostate cancer (PCa) is the second most diagnosed cancer of men in the world. In 2012, the newly diagnosed PCa cases estimated to be about 1.1 million cases. In developed countries, PCa is the first incident cancer while it is in the fourth place in developing countries with about 353,000 new cases in 2012. In terms of mortality, PCa is the third in developed countries and the sixth ranked in developing countries with about 165,500 deaths in 2012(2). The incidence and prevalence rates of PCa in the world are presented in Table 1. 

In 2015 the number of new case of PCa in Iran was estimated about 4260 with a five-year prevalence of more than 10000 patients (3,4). Prostatic neoplasm is the third most frequently diagnosed visceral cancer among men in Iran (7.75% of all new cancer cases). It is also the fourth reason of cancer-caused mortality in men in Iran. The annual incidence of PCa in Iran (age-adjusted by world standard population) is about 12.59 per 100,000 men, according to 2009 Iran Ministry of Health (MOH) cancer registry book. Incidence rate of this cancer is even higher in capital city of Iran (Tehran) with 22.72 cases per 100,000 men and it is the first frequently diagnosed cancer in men after non-melanoma skin cancer in this city. In comparison with previous released data, PCa shows an increase in incidence rate (Table 2) (5).

PCa is also a principal cause of cancer-related death in men (2). The Disability Adjusted Life Years (DALYs) related to PCa was estimated around 4.8 million globally in 2013, from which 43% was occurred in developing countries, and 57% was in developed countries (6).

*Castrate-Resistant Prostate Cancer*

“Castrate-resistant prostate cancer (CRPC) is defined by disease progression despite androgen depletion therapy (ADT) and may be presented as either a continuous rise in serum prostate-specific antigen (PSA) levels, the progression of pre-existing disease, and/or the appearance of new metastases”(7). When PSA rises whilst patient is under ADT and symptoms of disease progression is proofed with bone scanning and CT scanning, CRPC would be possible diagnosis. This stage of PCa consists of wide range of severity including PSA rise with no metastases nor symptoms to a very severe state with metastases to the bones and other tissues (7). CRPC is also the most challenging stage of PCa in terms of treatment strategies. Oncologists and Uro-oncologists have to decide on various options based on patients and tumor characteristics and it is one of the most complicated situations that is medical decision making. In recent years, various treatment options including extensive mechanisms of action have been introduced. Abiraterone acetate, enzalutamide, cabazitaxel, immunotherapy with sipuleucel-T, radionuclide therapy, and bone-targeted therapies (zoledronic acid, denosumab) are the main therapeutic options (8). The new treatment options will prolong the survival of patients and consequently their use of health care resources will increase dramatically. Therefore, the diagnostic and therapeutic costs of CRPC will bring about a significant economic burden in near future (9). 

*Aim of the Study:* Considering the challenges in the selection and sequencing of the best treatment options and the potential economic burden especially in a developing country with limited resources, the aim of this article is to review the clinical practice guidelines for the management of CRPC and to summarize the recommendations of these guidelines. Furthermore, clinical studies of the existing and emerging medicines will be reviewed to prepare a brief summary of their potential benefits as well as safety concerns. Some suggestions will also be prepared while keeping in mind the economic limitations in health resources in addition to the concept of Health-Related Quality of Life (HRQoL) and patient satisfaction.

## Methods

The PubMed and Scopus database were systematically searched and the relevant articles and guideline reviews were selected for scrutiny. PubMed database was searched using MeSH database with the following key-words: “Practice Guideline”[Publication Type], “Prostatic Neoplasms”[Mesh] , and (“2006/07/13”[PDat]: “2016/07/09”[PDat]). The documents that had been published since 10 years before the search date, including 98 articles, were reviewed for finding the relevant papers. In addition, Scopus database was searched using the following key-word combinations: ( TITLE-ABS-KEY ( guideline ) , TITLE-ABS-KEY ( “prostate cancer” ) , TITLE-ABS-KEY ( “treatment” or “management” ) , TITLE-ABS-KEY ( “castration resistant” or “castration-resistant” or “castrate-resistant” or “castrate resistant” or “hormone resistant” or “hormone-resistant” or “hormone-refractory” or “hormone refractory” or “hormone-insensitive” or “hormone insensitive” ) ). 

The search result included 203 articles. When time limitation was applied (published articles since 2007), 179 articles were accessed. By reviewing the titles and abstracts, 52 relevant articles were found. Since many articles were discussing the same guidelines and considering the last update of each guideline, the authors selected eight leading clinical guidelines from different health care settings among the most and less developed countries. The authors summarized treatment recommendations of each guideline for various risk-groups of patients. 

When the most recommended treatment options were identified, the main clinical trials on them were found within PubMed and the Cochrane library. A review on the papers was performed and the results were summarized focusing on overall survival, progression-free survival, HRQoL, time to progression, time to skeletal-related events, and other efficacy and safety outcomes.

*Guidelines review*

The practice guidelines by eight national and international societies and organizations were reviewed which are as follows: American Urological Association (AUA), National Comprehensive Cancer Network (NCCN), European Association of Urology (EUA), Spanish Oncology Genitourinary Group (SOGG), Asian Oncology Summit, Saudi Oncology Society-Saudi Urology Association combined guideline, National Institute for Health and Care Excellence (NICE), and Canadian Urological Association-Canadian Urologic Oncology Group (CUA-CUOG). 

*American Urological Association *(10,11)

The final AUA guideline for the treatment of CRPC was published in May 2013 and was updated in April 2014 and then in March 2015 to incorporate relevant newly published literature to provide a better rational basis for the management of patients. (Table 3). 

*Patient Classification*

In the AUA guideline, six categories of patients are defined representing the most common scenarios that are faced in clinical practice. These patients groups are categorized based on the presence or absence of metastatic disease, the degree of symptoms, the performance status of patients (defined by the ECOG scale), and their previous history of chemotherapy with docetaxel. Index Patient 1: Asymptomatic non-metastatic CRPC.

- Index Patient 2: Asymptomatic or minimally-symptomatic mCRPC, good performance status, no prior docetaxel chemotherapy.
- Index Patient 3: Symptomatic mCRPC, good performance status, no prior docetaxel chemotherapy.
- Index Patient 4: Symptomatic mCRPC, poor performance status, no prior docetaxel chemotherapy.
- Index Patient 5: Symptomatic mCRPC, good performance status, prior docetaxel chemotherapy.
- Index Patient 6: Symptomatic mCRPC, poor performance status, prior docetaxel chemotherapy.

*2. Canadian Urological Association- Canadian Urologic Oncology Group *(12) (Table 4). 

*3. The guideline proposed by Asian Oncology Summit *(13) (Table 5). 

*4. Spanish Oncology Genitourinary Group *(14,15) (Table 6). 

*5. National Institute for Health and Care Excellence (NICE) *(16–20) (Table 7). 

*6. European Association of Urology *(22–24) (Table 8). 

*7. Saudi Oncology Society and Saudi Urology Association *(25) (Table 9). 

*8. The US National Comprehensive Cancer Network *(26) (Table 10). 

NCCN Clinical Practice Guidelines in Oncology are used extensively in many health care systems even in low and middle-income countries. The NCCN Framework^™^ has established to define applicable treatment pathways that are suited with available resources. The categories of contexts are defined as basic, core, and enhanced levels. In this paper, the recommendations for systems with basic level of resources are presented. “Basic Resources” is defined as a level which “includes essential services needed to provide basic minimal standard of care” (27).

*Cytotoxic Medicines*

*Docetaxel*

The phase 3 TAX327 study compared docetaxel+prednisone versus mitoxantrone plus prednisone, and the results showed 2·4 month median prolongation in survival (HR 0·76, 95% CI 0·62–0·94; *p* = 0·009). Quality of life was measured using FACT-P tool in more than 800 patients and the score was also significantly improved with docetaxel specifically in prostate-specific subscale (28). The phase 3 SWOG-9916 trial compared docetaxel+estramustine with mitoxantrone+prednisone. The median overall survival was longer in the docetaxel group than in the group given mitoxantrone and prednisone (17.5 months vs. 15.6 months, *P* = 0.02) but pain relief was similar in both groups. High grade neutropenic fevers, nausea and vomiting, and cardiovascular events were more common among patients receiving docetaxel. (29). An extended survival analysis of TAX324 trial proved that hat survival of men with mCRPC is significantly longer after treatment with docetaxel+prednisone than with mitoxantrone+prednisone arm. Median survival time was 19.2 months (95% CI, 17.5 to 21.3 months) in the docetaxel arm and 16.3 months (95% CI, 14.3 to 17.9 months) in the mitoxantrone group. More patients survived ≥ 3 years in the docetaxel-receiving patients (16.6% -18.6%) compared with the mitoxantrone arm (13.5%) (30).

In Cochrane review of 47 RCTs on chemotherapy for hormone resistant PCa patients, 6929 patients were included. Drug categories included in this review were estramustine, 5-fluorouracil, cyclophosphamide, doxorubicin, mitoxantrone, and docetaxel. Although the improvement was less than 2.5 months, only docetaxel studies reported a significant improvement in overall survival compared to best standard of care. The mean percentage of patients achieving at least a 50% reduction in PSA compared to baseline was 48% with estramustine, 20% with 5-fluorouracil, 33% with mitoxantrone, 52% with docetaxel and 50% with the only one study on doxorubicin. Pain relief was reported in 35% to 76% of patients receiving either single agents or combination regimens. All cytotoxic treatments were associated with toxicity including mainly myelosuppression, gastrointestinal and cardiac toxicities, neuropathy, and alopecia (31).

*Cabazitaxel*

In the open-label randomized phase 3 TROPIC trial, 755 men with mCRPC who had received but failed to previous docetaxel therapy, were randomized to receive either 12 mg/m2 mitoxantrone along with 10 mg oral prednisone or 25 mg/m2 cabazitaxel along with prednisone. In the cabazitaxel group median survival was 15·1 months (95% CI 14·1–16·3) versus 12·7 months (11·6–13·7) in the mitoxantrone group. The hazard ratio of death for men treated with cabazitaxel was 30% lower compared with mitoxantrone cohort (95% CI 0·59–0·83, *p* < 0·0001) and median progression-free survival was 1.4 months higher in the cabazitaxel group. The incidence of clinically important adverse events were significantly higher with cabazitaxel compared to mitoxantrone group (neutropenia [82% vs. 58%], diarrhea [6% vs < 1%], febrile neutropenia 8% vs. 1%]) (32).

To assess the safety profile and health related quality of life for mCRPC patients treated with cabazitaxel, 112 patients from 12 cancer centers in UK were planned to receive cabazitaxel for 10 cycles. These patients had received docetaxel but showed disease progression before starting cabazitaxel. QoL was recorded at alternate cycles using the EQ-5D-3L questionnaire and visual analogue scale (VAS). Both QoL and VAS scores were improved from 0.7 to about 0.8 but no statistical analysis was performed to prove the significance (33). 

*Anti-Androgens*

*Abiraterone Acetate*

The efficacy of abiraterone acetate was proved in two landmark controlled trials COU-AA-301 and COU-AA-302 in which abiraterone was tested on mCRPC patients with or without prior docetaxel therapy respectively. 

The COU-AA-301 study enrolled 1195 patients at 147 sites in 13 countries. Eligible patient had mCRPC progressing after docetaxel. Patients received either 1000 mg abiraterone acetate once a day plus 5 mg prednisone BD or placebo plus prednisone. The primary endpoint was overall survival. At median follow-up, patients on abiraterone had 4.4 months higher overall survival than placebo group. Median time to PSA progression was also significantly longer with abiraterone. The most common grade 3–4 adverse events including fatigue, anemia, and bone pain did not differ significantly between groups and the incidence rate ranged between 6-10 percent of patients in both groups (34). In patients with clinically significant pain at baseline, abiraterone acetate resulted in significantly more palliation than prednisone alone (40% of patients vs. 28.8% of patients). Significantly faster palliation (median time to palliation 5·6 months vs. 13·7 months, p = 0·0018) of pain intensity was resulted by abiraterone than with prednisone alone. Median time to occurrence of first skeletal-related event was significantly longer (25 months vs. 20.3 months) with abiraterone acetate and prednisone than with prednisone alone (35). Along with the demonstrated survival benefit for abiraterone, HRQoL improvement and delay in HRQoL worsening was likewise in favor of Abiraterone group. Abiraterone resulted in significantly better FACT-P outcomes than prednisone, with the exception of the Social/Family Well-Being (SFWB) subscale. Significant improvements in the FACT-P total score were observed in 48% of patients receiving abiraterone versus 32% of patients receiving prednisone (p < 0.0001) in COU-AAA-301 trial (36). Abiraterone acetate provides significant clinical benefit in terms of improvements in OS and PSA response rates, post-docetaxel therapy, in patients either with or without baseline visceral disease (37). In mCRPC patients with previous docetaxel chemotherapy, abiraterone acetate improved patient-reported fatigue and time to fatigue improvement compared with prednisone alone. These results were statistically significant and clinically meaningful (38). 

In the COU-AA-302 study, 1088 patients were randomly assigned to receive abiraterone acetate plus prednisone or placebo plus prednisone. The median radiographic progression free survival (PFS) was 16.5 months in abiraterone group and 8.3 months in placebo group (*P *< 0.001). After a median follow-up time of 22.2 months, overall survival was improved significantly with abiraterone. Additionally, abiraterone significantly delayed the initiation of chemotherapy and opiate use compared to prednisone alone (39). Since, PFS in metastatic mCRPC trials has not been defined consistently and has poor association with overall survival (OS), a reproducible quantitative definition of radiographic PFS (rPFS) was tested for association with the primary end point of OS in a COU-AA-302 trial. rPFS was highly consistent and highly associated with OS, providing initial prospective evidence on further developing rPFS as an intermediate end point in mCRPC trials (40). With a median follow-up duration of 27.1 months, rPFS improvement was significantly higher with abiraterone versus prednisone (median: 16.5 vs. 8.2 months; *p* < 0.0001). Abiraterone improved OS (median: 35.3 vs. 30.1 months; *p* = 0.0151) but this survival time did not reach the pre-specified efficacy level (41). Median time to progression to median pain intensity, pain interference with daily activities as well as median time to progression of worst pain were longer with abiraterone vs. prednisone alone. All the differences in time to progression of pain were significant except the latter. Median time to HRQoL score deterioration was also significantly longer in abiraterone group (42). In a subgroup analysis of men aged 75 years and older, abiraterone acetate was proved to have clinical benefit and to be well tolerated in elderly and younger men with chemotherapy naïve mCRPC. The subgroup analysis support the use of abiraterone in elderly patients who may not tolerate other therapeutic options with higher toxicity (43).

*Enzalutamide*

The efficacy and safety of enzalutamide was established in two large randomized controlled trials which were performed on near 3000 metastatic hormone resistant patients with PCa before and after chemotherapy. The first phase III clinical trial (the AFFIRM study) was published in 2012 presenting mainly the overall survival benefit on the patient post docetaxel therapy. The primary analysis of the other leading clinical trial (the PREVAIL study) was published in 2014 focusing on radiographic progression-free survival and overall survival as co-primary endpoints. 

AFFIRM (A Study Evaluating the Efficacy and Safety of the Investigational Drug MDV3100) was a multi-center phase III RCT on enzalutamide in mCRPC patients who had failed or progressed after chemotherapy. From 156 centers in 15 countries 1199 patents were randomly assigned, in a 2:1 ratio, to receive oral enzalutamide 160 mg/day (800 patients) or placebo (399 patients). The median overall survival was 18.4 months in the enzalutamide cohort versus 13.6 months in the control group (HR: 0.63, *P* < 0.001). Enzalutamide was also significantly superior over placebo in secondary endpoints. In enzalutamide group 50% of patients showed at least 50% reduction in PSA level by 50% versus 2% of patients in placebo group (*P* < 0.001). Quality-of-life response rate defined as at least 10 point improvement in global score of FACT-P was significantly better with enzalutamide (43% vs. 18%, *P* < 0.001). Enzalutamide resulted in about 5.3 months longer time to PSA progression compared to placebo. Radiographic progression-free survival was also longer with enzalutamide (8.3 vs. 2.9 months; hazard ratio, 0.40; *P* < 0.001). Additionally, patients on enzalutamide had lower risk to show the first skeletal-related events (16.7 vs. 13.3 months; hazard ratio, 0.69; *P* < 0.001). Adverse events including fatigue, diarrhea, and hot flashes were more frequent in the enzalutamide group and seizure was reported in 0.6% of patients on enzalutamide (44). Exploratory analysis to assess the efficacy outcomes based on differences in patient characteristics specifically the baseline PSA level in the AFFIRM trial, demonstrated that benefits in overall survival and time to PSA progression with enzalutamide is not related to the baseline disease severity (45). Another post hoc analysis of the AFFIRM trial showed that pain palliation, median time to pain progression and median time to HRQoL score deterioration were significantly improved with enzalutamide versus placebo (46). A post hoc subgroup analysis of elderly patients (≥ 75 years) in the AFFIRM study proved the similar efficacy, safety and tolerability of enzalutamide in both subgroups of younger and older patients (47). 

In the PREVAIL trial 1717 patients with metastatic PCa who have not received chemotherapy were randomly assigned in two cohorts to receive either enzalutamide 160 mg/day or placebo. Enzalutamide decreased the rate of rate of radiographic progression-free survival 81% relative to placebo (*P* < 0.001). At the cut-off date 72% of patients n enzalutamide were alive compared to 63% in control group (HR of death: 0.71, *P* < 0.001). Enzalutamide was also significantly superior against placebo in all secondary endpoints including the lag time until the progression to the use of chemotherapy, the time until the first skeletal-related event, a complete or partial soft-tissue response, and the time to PSA progression ratio. The PSA decline of at least 50% was observed in 78% of patients in enzalutamide group vs 3% of control patients (48). HRQoL was assessed using FACT-P and EQ-5D and pain was assessed using Brief Pain Inventory Short Form (BPI-SF) in the PREVAIL study. Median time to deterioration in FACT-P total score was 5.7 months longer compared to placebo and 40% of patients in treatment group versus 23% of control patients reported clinically important improvements in FACT-P total score. Median time to progression in BPI-SF pain at its worst also differed significantly in favor of enzalutamide but it was not clinically meaningful (49).

*Immunotherapy*

*Sipuleucel-T*

“Sipuleucel-T is an autologous cellular immunotherapy for asymptomatic/minimally symptomatic metastatic castrate-resistant prostate cancer (CRPC)” (50). Sipuleucel-T is the first vaccine for treatment of which received FDA approval (51). 

The first phase III trial on sipuleucel-T was a placebo-controlled on 127 patients with asymptomatic mCRPC who randomly received three infusions of sipuleucel-T or placebo twice monthly. The patients were followed for 36 months for survival assessment. Median survival with sipuleucel-T was 25.9 months for and 21.4 months for placebo (*P* = 0.01) with a not significant time to disease progression compared to placebo (11.7 vs. 10 weeks, *P* = 0.052) (52). 

The same protocol was performed in another phase III RCT on 512 patient (IMPACT study). The recruited patients had an expected survival of at least 6 months. Patients were recruited since August 2003 until November 2007 and by January 2009, 22% reduction in the risk of death was perceived with sipuleucel-T (HR: 0.78; *P* = 0.03). The medial survival was 25.8 vs. 21.7 months but the time to objective disease progression was similar in both groups. Additional therapies after intervention period included docetaxel use which slightly higher in sipuleucel-T group (57% vs. 50%) and the Kaplan–Meier estimate of the median time to docetaxel use was 1.6 months earlier in treatment group. The most frequent adverse events in sipuleucel-T group included chills, fever, headache, influenza-like syndrome, myalgia, hypertension, hyperhidrosis, and groin pain which most of them were improved within 1-2 days (53). 

*Radiopharmaceuticals*

*Strontium-89 and Samarium-153*

Strontium-89 and Samarium-153 are radioisotopes with β-emitting activity that received U.S. FDA approval for pain relief of bone metastatic CRPC patients (54). 

In a phase III randomized trial, strontium-89 with a single dose of 10.8 milicurie was compared to placebo in bony metastatic hormone resistant PCa patients who under treatment with local field radiotherapy. Analysis of the survival did not show any significant difference between strontium-89 and placebo. Progression of pain, which was measured by the number of new sites of pain or the necessity radiotherapy, and the Intake of analgesics for control of pain, decreased significantly in treatment group compared to placebo. Over the first four months, tumor markers including PSA and alkaline phosphatase were also reduced significantly in treatment group. Quality of life in terms of physical activity and pain alleviation showed significant improvement in favor of strontium-89. Hematologic toxicities were significantly higher in treatment group in terms of both white blood cell and platelet levels (55). In a systematic review on clinical trials of strontium-89 in the management of pain in the bony metastatic patients with prostate or breast cancer up to 80% of patients showed pain relief and about 10% got pain free. The severity of hemotoxicity was reported as mild in this review (56). In another phase trial of strontium-89 in patients with bone metastases due to prostate, breast, and other types of cancer, pain was considerably improved in 58% of patients in stronsium-89 group following by an improved quality of life (57).

In a phase III randomized trial for the efficacy evaluation of samarium-153 (153 Sm)-lexidronam, also referred as samarium-153 EDTMP, 152 men with CRPC and painful bone metastases were enrolled. Patients in two cohorts received either radioactive samarium-153 at a dose of 1 mCi/Kg or non-radioactive samarium-152 both as lexidronam complex. Statistically significant Improvement on measures of pain compared with control within the first 2 weeks and opioid use reduction at weeks 3 to 4 was reported. The only adverse event relating to samarium was mild and transient bone marrow suppression. WBC and platelet counts recovered to baseline at approximately 8 weeks (58). In a clinical trial on 35 patients with bone metastasis arising from various tumor types, pain palliation was observed 80% of the patients and 54% of them reported substantial or complete pain relief. Moderate myelosuppression was reported in one patient (59).

A comparative trial of strontium-89 and samarium-153 proved that the similar pain relief with both radiopharmaceuticals in both prostate and breast cancer patients. The frequency of severe adverse events were also reported as rare with both comparators (60).

*Radium-223*

Radium-223 chloride formerly known as alpharadin is a first-in-class α-particle-emitting radiopharmaceutical, which provides survival benefit for patients with hormone-resistant prostatic neoplasm which has spread to the bones (61). Radium-223 is a calcium mimetic element with preferential uptake in bone mineral hydroxyapatite. It targets tumor cells near the areas on new bone formation. Ra223 forms complexes with hydroxyapatite and consequently gets integrated in the bony matrix (62). The novel mechanism of action of Ra223 brings about low rates of hematologic adverse events and makes it a potential treatment option in many of symptomatic mCRPC patients before docetaxel challenge (63). The approval of Ra223  in May 15, 2013 by the U.S. Food and Drug Administration (FDA) was based on a randomized, placebo-controlled, international trial (the “ALSYMPCA” trial) (54). 

In the ALSYMCA study, 921 castration resistant patients with two or more bone metastases detected on skeletal scintigraphy and no known visceral metastases were recruited. Patients were randomly assigned to receive either Ra223 or placebo along with best standard care. The patients received six periods of Ra223 with dose of 50 kBq/Kg (1.35 microcurie/Kg) or similar placebo as intravenous injection every four weeks. The planned follow-up time was up to 3 years. At the end of study period, overall survival with Ra223 was 3.6 months longer than with placebo along with 30% lower risk of death (median, 14.9 months vs. 11.3 months; HR: 0.70; *P* < 0.001). Radium-223 also prolonged the time to the first symptomatic skeletal event about 5.6 months relative to placebo (median, 15.6 months vs. 9.8 months; HR: 0.66; *P* < 0.001). The times to increase in alkaline phosphatase level as well as PSA level were also significantly prolonged with Ra223. The proportion of patients who had at last one adverse event were similar in both groups (93% vs 96%) and generally no clinically meaningful differences were observed between cohorts in terms of the frequency of grade 3 or 4 adverse events.  The treatment seems to be as safe as placebo but the only one report of grade 5 thrombocytopenia in a patient in the treatment group and considered to be related to Ra223. In terms of HRQoL, a higher proportion of patients in treatment group had a meaningful improvement in the FACT-P total score, during the intervention period (25% vs. 16%, *P* = 0.02) (64). The subgroup analysis of ALSYMCA trial for patients with or without previous docetaxel therapy proved the efficacy of Ra223 in both subgroups in terms of overall survival and most of the secondary endpoints. The safety profile however was in favor of patients without previous docetaxel therapy. Patients in Ra223 group and with previous docetaxel therapy had a higher incidence of grade 3-4 thrombocytopenia (65).

*Bone protecting agents*

*Denosumab and Zoledronic Acid*

Denosumab was compared with zoledronic acid in phase 3 double blind study in men with CRPC metastatic to the bone with no previous exposure to intravenous bisphosphonate. In this multi-center trial, 1904 patients were randomized to receive either denosumab or zoledronic acid in 1:1 ratio. Patients received 120 mg denosumab subcutaneously plus intravenous placebo, or 4 mg intravenous zoledronic acid along with a subcutaneous placebo, Q 4 weeks. Median time to the first skeletal-related event (considering study duration only) was 20·7 months in denosumab group versus 17·1 months in zoledronic acid group (HR for the first and subsequent skeletal-related events: ·82, p = 0·0002 for non-inferiority; p = 0·008 for superiority). More events of hypocalcaemia occurred in the denosumab group (121 [13%]) than in the zoledronic acid group (55 [6%]; *P* < 0·0001). Osteonecrosis of the jaw occurred infrequently (22 [2%] vs. 12 [1%]; p = 0·09). The rate more frequent adverse events including anemia, back pain, decreased appetite, nausea, fatigue, constipation, bone pain, asthenia, arthralgia, severe pain and peripheral edema ranged between 18 to 36 percent in both groups with no significant difference. The incidence rate of any adverse events was 97% in each group in addition to no significant difference in serious and fetal adverse events (about 61.5% and 29.5% respectively). Furthermore, the rate of grade 3 or 4 adverse events (72% vs. 66%) and hypocalcemia (13% vs. 6%) were significantly higher with denosumab (66). 

**Table 1:** Incidence and Prevalence of Prostate Cancer in the World

MORE DEVELOPED AREAS				LESS DEVELOPED AREAS			
INCIDENCE		MORTALITY		INCIDENCE		MORTALITY	
**ASR**	Cumulative risk, % (aged birth to 74 years)	ASR	Cumulative risk, % (aged birth to 74 years)	ASR	Cumulative risk, % (aged birth to 74 years)	ASR	Cumulative risk, % (aged birth to 74 years)
**69.5**	8.8	10	0.8	14.5	1.7	6.6	0.6

ASR: Age Specific Rate. (Source: Torre, 2015)

**Table 2:** . Incidence Rate of Prostate Cancer in Iran

Year	2005-2006 (1384)	2006-2007 (1385)	2007-2008 (1386)	2008-2009 (1387)	2009-2010 (1388)
**Prostate Cancer ASR**	9.22	9.57	10.91	12.8	12.5

Source: cancer Registry Book 2009, Iran Ministry of Health, Cancer office

**Table 3:** American Urological Association (10,11).

Index Patient number	Situation	Recommendation/Option/Standard		Evidence Level Grade
Index Patient 1	Asymptomatic non-metastatic CRPC	Observation with continued Androgen Deprivation Therapy [Recommendation]		C
	Patients unwilling to accept observation	1^st^ generation antiandrogens (Flutamide, Bicalutamide, Nilutamide) or 1^st^ generation Androgen Synthesis Inhibitors (Ketoconazole+Steroid) [Option]		C
	patients outside the context of a clinical trial	Systemic chemotherapy or immunotherapy should not be used [Recommendation]		C
Index Patient 2	---	Abiraterone + Prednisone, Enzalutamide / Docetaxel, or Sipuleucel-T [Standard]		A/B
	Patients who do not want or cannot have one of the standard therapies	1^st^ generation Anti-androgen therapy, Ketoconazole + Steroid or observation [Option] Manipulation with existing antiandrogen agents, such as Bicalutamide, Nilutamide or Flutamide [Option]		C
Index Patient 3	---	Abiraterone + Prednisone, Enzalutamide / Docetaxel [Standard]		A/B
	Patients who do not want or cannot have one of the standard therapies	Ketoconazole + Steroid / Mitoxantrone / Radionuclide therapy [Option]		C/B/C
	Patients with symptoms from bony metastases and without known visceral disease	^223^Ra(Radium) [Standard]		B
	---	Estramustine or sipuleucel-T should not be used [Recommendation]		C
Index Patient 4	---	Abiraterone + prednisone or enzalutamide [Option]		C
	Patients who are unable or unwilling to receive abiraterone + prednisone or enzalutamide	Ketoconazole+ steroid or radionuclide therapy [Option]		C
	Select cases, specifically when the performance status is directly related to the cancer	Docetaxel or Mitoxantrone [Expert opinion]		-
	patients with symptoms from bony metastases and without known visceral disease	^223^Ra(Radium) (specifically when the performance status is directly related to symptoms related to bone metastases) [Expert opinion]		-
	---	sipuleucel-T should not be used [Recommendation]		C
Index Patient 5	---	Abiraterone + Prednisone / Cabazitaxel / Enzalutamide [Standard] If the patient received Abiraterone + Prednisone prior to Docetaxel chemotherapy, they should be offered Cabazitaxel or Enzalutamide		A/B/A
	If Abiraterone + Prednisone, Cabazitaxel or Enzalutamide is unavailable	Ketoconazole + Steroid [Option]		C
	Patients who were benefitting at the time of discontinuation (due to reversible side effects) of Docetaxel chemotherapy	Retreatment with Docetaxel [Option]		C
	patients with symptoms from bony metastases and without known visceral disease	^223^Ra(Radium) [Standard]		B
Index Patient 6	---	**Palliative care**: Alternatively, for selected patients, treatment with Abiraterone + Prednisone, Enzalutamide, Ketoconazole + steroid or Radionuclide therapy may be offered [Expert opinion]		-
	---	Systemic chemotherapy or immunotherapy should not be used [Expert opinion]		-
Bone Health	Patients with fractures and skeletal related events to CRPC	Preventative treatment (e.g. supplemental calcium, vitamin D) [Recommendation]	C		
	mCRPC patients with bony metastases	Denosumab or Zoledronic acid as preventative treatment for skeletal related events [Option]	C		

Treatment given in the last months of life may delay access to end of life care, increase costs and add unnecessary symptom management. Patients with poor performance status (ECOG 3 or 4) should not be offered further treatment.

**Table 4:** Canadian Urological Association-Canadian Urologic Oncology Group (12).

CRPC type	Patient situation	Recommendation		Level/Grade
Non-metastatic	Rising PSA	No approved regimen and no standard of care exists. Discontinuation of AA therapy should be considered if patients are receiving these agents. Secondary hormonal treatments (excluding Abiraterone or Enzalutamide) may be attempted.		3/C
Metastatic	without symptoms or minimally symptomatic	Introduction of, or changes to, a first-generation AA or the use of corticosteroids with or without Ketoconazole		3/C
		Abiraterone acetate 1000 mg/day + Prednisone 5 mg bid is recommended as first-line therapy		1/A
		Enzalutamide 160 mg/day is recommended as first-line therapy		1/A
		Treatment with Docetaxel 75 mg/m^2^ q3W + 5 mg oral Prednisone bid		1/A
	With symptoms	Treatment with Docetaxel 75 mg/m^2^ q3W + 5 mg oral Prednisone bid is recommended		1/A
		For patients with pain due to bone metastases and who do not have visceral metastases ^223^Ra(Radium) q4W for 6 cycles is recommended		1/A
		For patients who cannot receive or refused Docetaxel, combination of Abiraterone acetate 1000 mg/day + Prednisone 5 mg bid or Enzalutamide 160 mg/day should be considered as first-line therapy		(Expert opinion)
	Patients who progress after Docetaxel-based chemotherapy	Proved survival benefit	Cabazitaxel (25 mg/m^2^) + Prednisone (5 mg/day)	1/A
			Abiraterone acetate (1000 mg per day) + Prednisone (5 mg bid)	1/A
			Enzalutamide (160 mg/day)	1/A
			^223^Ra(Radium) q4W for 6 cycles	1/A
		unknown survival benefit	Docetaxel + Prednisone re-exposure in patients who have had a previous favorable response to Docetaxel may be reasonable	(Expert Opinion)
			For palliative pain relief Mitoxantrone + Prednisone may be offered (Grade C).	C
	Patients with CRPC and bone metastases	Denosumab (120 mg subcutaneous) or Zoledronic acid (4 mg IV) q4W, along with daily calcium and vitamin D supplementation		1/A

**Table 5:** Asian Oncology Summit (13).

CRPC type	Patient situation	Recommendation
Non-metastatic	Rising PSA	Non-steroidal Anti-Androgens / Ketoconazole
Metastatic	---	Docetaxel (as an standard first-line therapy) Docetaxel + Prednisone or Mitoxantrone + Prednisone can be used
	Progression after Docetaxel-based chemotherapy	Cabazitaxel as an cytotoxic agents
		Abiraterone acetate (it is 10 times more potent than Ketoconazole in this regard)
		Concurrent Prednisone should be considered
		Enzalutamide, ^223^Ra (Radium) and sipuleucel-T
Palliative approaches		
Bone protection	To reduce skeletal-related events In patients with metastatic CRPC, Zoledronate and denosumab should be used.	
Chemotherapy	In palliating metastatic CRPC patients Mitoxantrone + Prednisone is effective.	
	Radionuclide Therapy by the strontium (^89^Sr) as a calcium mimetic preferentially taken into sites of osteoblastic disease can be used.	
	Palliative surgical interventions such as channel transurethral resection of prostate or ureteric stenting can be used.	

For countries with enhanced level of resources, palliative chemotherapy with Docetaxel and Cabazitaxel, and Bone protection with Zoledronic acid or Denosumab are recommended.

Third-line hormone therapy—e.g., abiraterone, Enzalutamide, or Ketoconazole and Bone-seeking α-particle therapy or radioisotope therapy and Palliative chemotherapy with Docetaxel and Cabazitaxel, and Bone protection with Zoledronic acid or Denosumab are recommended for countries with maximum level of resources(13).

**Table 6:** Spanish Oncology Genitourinary Group (14,15).

CRPC type	Patient situation	Recommendation	Level/Grade
Without metastases or symptoms	With rising PSA	LHRH analogs should be continued in patients with CRPC	3/C
	Antiandrogen withdrawal	Anti-androgen withdrawal should be considered in patients with CRPC (except in symptomatic patients or in patients who have a rapid and aggressive progression).	2b/B
Metastatic CRPC	Asymptomatic or minimally symptomatic patients	As an option Ketoconazole + Hydrocortisone and Anti-androgen withdrawal in asymptomatic CRPC produces a better response than Anti-androgen withdrawal alone.	2b/B
		Sipuleicel-T (before chemotherapy with Docetaxel)	1b/A
		Abiraterone for patients without visceral metastases and previously untreated with chemotherapy	1b/A
		Enzalutamide for selected patients with visceral metastases, who have not received previous chemotherapy	1b/A
		Patients with adverse prognostic factors (presence of visceral metastases) should also be considered for Docetaxel treatment	1a/A
	Symptomatic patient and/or with visceral metastases	Docetaxel (75 mg/m^2^ q3W) + Prednisone (5 mg bid) as a standard first-line chemotherapy	1a/A
		Asymptomatic patients with mCRPC might be treated with the same Docetaxel schedule, particularly if additional factors of poor prognosis are present	1a/A
	Patients who progress after Docetaxel chemotherapy	Docetaxel rechallenge should be used only for patients who progressed after Docetaxel response and who did not experience any severe toxicity.	-
		In patients with symptomatic bone metastases and without visceral metastases, after Docetaxel or in those patients who are not eligible for chemotherapy ^223^Ra (Radium) is a reasonable treatment option.	1b/A
		Treatment with Abiraterone should be considered for patients with mCRPC following progression with Docetaxel	1b/A
		Cabazitaxel should be considered for the treatment of patients with mCRPC with progressive disease after Docetaxel-based treatment	1b/A
		Alternative treatments after Docetaxel and/or Cabazitaxel and/or Abiraterone include Docetaxel rechallenge, Mitoxantrone, oral Cyclophosphamide or Vinorelbine chemotherapy	2b/B

**Patients with bone metastases: Bone targeted therapies**

**Table 7:** National Institute for Health and Care Excellence (NICE) (16–20).

Type of prostate cancer	Recommendation
Metastatic prostate cancer	Offer bilateral orchiectomy to all men with metastatic prostate cancer as an alternative to continuous LHRH agonist therapy.
	Anti-androgen monotherapy with Bicalutamide (150 mg) can be offered in men with metastatic PCa who are willing to accept the adverse impact on overall survival and gynaecomastia in the hope of retaining sexual function.
	Begin androgen deprivation therapy and stop Bicalutamide treatment in men with metastatic PCa who are taking Bicalutamide monotherapy and who do not maintain satisfactory sexual function.
Hormone-relapsed metastatic prostate cancer	Enzalutamide is recommended, as an option for treating metastatic hormone relapsed prostate cancer, in people who have no or mild symptoms after androgen deprivation therapy has failed, and before chemotherapy is indicated.
	Docetaxel as a treatment option for men with hormone-refractory prostate cancer is recommended only if their Karnofsky performance-status score is 60% or more.
	^223^Ra (Radium) as an option for treating adults with hormone relapsed PCa, symptomatic bone metastases and no known visceral metastases is recommended only if they have had treatment with Docetaxel.
	Corticosteroids such as Dexamethasone (0.5 mg daily) as third-line hormonal therapy after androgen deprivation therapy and anti-androgen therapy could be used.
	Abiraterone in combination with Prednisone or Prednisolone is recommended as an option for the treatment of mCRPC in adults, only if their disease has progressed on or after one Docetaxel-containing chemotherapy regimen.
	Cabazitaxel in combination with Prednisone or Prednisolone is recommended as an option for patients whose disease has progressed during or after Docetaxel chemotherapy, only if: has an eastern cooperative oncology group (ECOG) performance status of 0 or 1 has had 225 mg/m2 or more of Docetaxel treatment with Cabazitaxel is stopped when the disease progresses or after a maximum of 10 cycles (whichever happens first)
**Bone-targeted therapies** Do not offer Bisphosphonates to prevent or reduce the complications of bone metastases in men with hormone-relapsed PCa. Bisphosphonates for pain relief may be considered for men with hormone-relapsed PCa when other treatments (including analgesics and palliative radiotherapy) have failed. Choose the oral or IV route of administration according to convenience, tolerability, and cost. Strontium-89 should be considered for men with hormone-relapsed PCa and painful bone metastases, especially those men who are unlikely to receive myelosuppressive chemotherapy	

Marketing authorization for sipuleucel-T was withdrawn on 19 May 2015 (21).

**Table 8:** European Association of Urology (22–24)

Treatment of CRPC	Recommendation
First-line treatment	Sipuleucel-T (Sip-T) as first line therapy is recommended (not approved by European Medicines Agency yet)
	Abiraterone acetate + Prednisone is approved for treatment of asymptomatic and mildly symptomatic mCRPC patients
	Docetaxel + Prednisone is also recommended as first line therapy for CRPC The most appropriate indication for chemotherapy might be the clinical scenario of symptomatic or extensive metastases, rapid PSA DT, high Gleason score, or short-term response to primary ADT
Second-line treatment	Docetaxel rechallenge for patients who might be good candidates for re-exposure
	Abiraterone acetate + Prednisone for progressive mCRPC patients who failed Docetaxel-based chemotherapy.
	Enzalutamide with 10 times greater affinity to the AR relative to Bicalutamide
	Cabazitaxel + Prednisone
Bone-targeting and bone-metastasis targeting agents	Zoledronic acid or Denosumab (The median time to first bone metastases will significantly be prolonged by denosumab). ^223^Ra (Radium) as a calcium mimic.

Currently, there is lack of evidence on a specific sequence of therapy. Therefore, physicians should adhere to the inclusion criteria of the various clinical trials when treating real-world patients with CRPC. Furthermore, the EAU guideline panel on PCa believes that any patient with PCa and especially CRPC is on a clinical trial.

**Table 9:** Saudi Oncology Society and Saudi Urology Association (25)

CRPC type	Patient situation	Recommendation
Non-metastatic	Patients who were on LHRH antagonist/agonists	These patients should continue LHRH antagonist/agonists indefinitely.
	With rising PSA	Secondary hormonal manipulations may be offered by either adding a Nonsteroidal anti androgen, Antiandrogen withdrawal, Ketoconazole, Steroids, diethylstilbestrol, or other estrogens
Metastatic	Asymptomatic	Abiraterone with Prednisone, systemic chemotherapy, or secondary hormonal manipulations (adding a non-steroidal antiandrogen, or antiandrogen withdrawal)
	Symptomatic	Abiraterone + Prednisone (only in mildly symptomatic patients) or systemic chemotherapy.
	patients with performance status 0-2 (by Eastern Cooperative Oncology Group scale)	Systematic chemotherapy in the form of Docetaxel + Prednisone should be offered only to these patients
	Patients who fail Abiraterone	Docetaxel + Prednisone.
	Patients who fail Docetaxel	Cabazitaxel with Prednisone, Abiraterone acetate (if not received in chemo-naive setting), or Enzalutamide. Patients who have disease limited to the bone can also be offered alpharadin (Radium 223) in addition.
	Patients with bony metastases	Denosumab therapy q4w (if not available Zoledronic acid can be given)

**Table 10:** The US National Comprehensive Cancer Network (26)

CRPC type	Patient situation	Recommendation
without Signs of Metastasis	-	Secondary hormone therapy (anti-androgen, anti-androgen withdrawal, corticosteroid, Ketoconazole with or without hydrocortisone, diethylstilbestrol or other estrogen) for patients with PSADT less than 10 months. Anti-androgen withdrawal should be offered to progressive patients while on combined androgen blockade.
Metastatic	Bone metastases	Zoledronic Acid or Denosumab is recommended
		For patients without visceral metastases, Radium-223 as a category 1 option to treat symptomatic bone metastases is recommended.
		Systemic radiotherapy using Samarium-153 or Strotium-89 for patients that the tumor does not respond to palliative chemotherapy or systemic analgesia and the patient is not a candidate for EBRT.
	Asymptomatic or Minimally Symptomatic	Sipuleucel-T is recommended for patients with good performance level (ECOG 0-1), estimated life expectancy more than 6 months and no liver metastases.
	No Visceral Metastases	Enzalutamide and Abiraterone + Prednisone as first line treatment options for asymptomatic, chemotherapy-naive patients with metastatic CRPC.
		Docetaxel + Prednisone can be used in asymptomatic patients when rapid progression or visceral metastases occur.
	Visceral Metastases	Docetaxel + Prednisone is the first line therapy for symptomatic metastatic patients (The addition of Estramustine is not recommended because it does not enhance efficiency and increases side effects)
		Enzalutamide is another category 1 recommendation
		Abiraterone is category 2 recommendation because of lack evidence in these patients
		Mitoxantrone is an option for patients who cannot tolerate Docetaxel
	Progression after Enzalutamide or Abiraterone	Docetaxel + Prednisolone is recommended as category 1
		Abiraterone for patients who have been on Enzalutamide and Enzalutamide for patients who have been on Abiraterone could be used
		Sipuleucel-T could be used if patient is asymptomatic or minimally symptomatic and without visceral or liver metastases.
	Progression after Docetaxel	No consensus exists but options include Abiraterone plus Prednisone, Enzalutamide, Radium (for symptomatic bone metastases without visceral metastases), Sipuleucel-T ( for patients with previously explained conditions), Docetaxel rechallenge, Secondary ADT, Cabazitaxel (for symptomatic patients with metastases) or Mitoxantrone (for patients who are not candidates for taxane-based chemotherapy)
	Post Cabazitaxel	Palliative care with prednisone or dexamethasone in low doses, Mitoxantrone but no other chemotherapy regimen

Some explanations about categorizations of evidences and levels of recommendations by guidelines are prepared in a supplementary file which is available at the journal webpage for this paper.

In a placebo controlled phase III RCT on 1432 non-metastatic CRPC patients at high risk of bone metastasis, denosumab significantly increased bone-metastasis-free survival (median 29·5 month vs. 25·2 months in placebo group; HR: 0·85, *P* = 0·028). Denosumab similarly delayed the time to first bone metastasis by 3.7 months; HR 0·84, p = 0·032). Overall survival did not differ between treatment and placebo group significantly. Rates of all adverse events and serious ones were nearly the same in both groups with no significant difference. Osteonecrosis of the jaw (5% vs. 0%) and hypocalcaemia (2% vs. < 1%) were significantly higher with denosumab (67). 

In a randomized, placebo-controlled, Phase III trial on 422 patients with CRPC, efficacy, and safety of zoledronic acid was compared with placebo. Zoledronic acid significantly palliated pain compared with placebo at 3, 9, 21, and 24 month. The annual incidence of skeletal-related events was also reduced by 49% with zoledronic acid. In patients without pain at the beginning of the study, zoledronic acid delayed the onset of bone pain compared with placebo (68). 

A pooled data analysis was performed on the three landmark double-blind phase III studies comparing denosumab with intravenous zoledronic acid in patients with bone metastases from breast cancer, castration-resistant PCa, or other solid tumors. The onset of moderate or severe pain was 6.5 months with denosumab compared to 4.7 months with zoledronic (HR: 0.83; p < 0.001). There was also 17% risk reduction for overall pain interference with denosumab compared to zoledronic acid. HRQoL score improvement, measured by FACT-G, did not show significant difference between treatments. Fewer patients (Absolute Difference: 0.9%-3.4%, Average Relative Difference: 4.1%) on denosumab experienced clinically important decrease from baseline in FACT-G total score in comparison with patients on zoledronic acid (P = 0.005) (69).

The TRAPEZE Randomized Clinical Trial was designed to determine the clinical effectiveness and cost-effectiveness of the combination of docetaxel, zoledronic acid, and Strontium 89, in CRPC metastatic to the bone in terms of bone symptom palliation and survival prolongation in case of docetaxel. A total of 757 were randomized to receive docetaxel alone or with zoledronic acid, Sr89, or both. Clinical PFS and overall survival was not significantly different with either Sr89 or ZA. Time to skeletal-related events was delayed significantly with zoledronic acid. Strontium-89 combined with docetaxel improved Clinical PFS but did not improve OS, SRE-free interval, or total SREs. Zoledronic acid reduced the risk of symptomatic skeletal events  by about 50% (70). 

## Discussion

We reviewed the core guidelines and clinical evidences used in treatment of patients suffering from CRPC. All of them are focusing on clinical efficacy without any economical effectiveness concerns. The pattern of treatments and selection of interventions are based on countriesꞌ regulation and health sector resources. 

This research found that in the field of Anti-Androgens, both enzalutamide and abiraterone are recommended in different stages of treatment by all of the guidelines. Clinical evidences showed their superior efficacy in comparison with their alternatives. These medicines are not accessible in Iran routinely, because of not listing in Iran Drug List.

Focusing on cytotoxic medicines, both docetaxel and cabazitaxel are recommended or suggested in different sequences of disease management. Although docetaxel is available in Iranian market, the newer alternative, cabazitaxel, is an expensive option in cases with treatment failure and it is not still officially accessible by patients.

Sipuleucel-T, which has received FDA approval for CRPC is also recommended by some guidelines but it is not accessible in many countries yet. One of the major hurdles for availability and accessibility of such treatments is that technological infrastructure behind the use of them is very costly and limited in many contexts. 

Radium-223 is strictly recommended in patients with bony metastases and its efficacy and safety is proofed through clinical trials. This option is not also available in Iran because of similar limitation which was mentioned for immunotherapy. However, the less effective and more hazardous alternative, samarium-153, is available in some nuclear medicine centers of Iran.

Finally, in the field of bone protecting agents, denosumab and zoledronic acid are recommended similarly by different guidelines. Although, the level of efficacy slightly favors denosumab, but many guideline except Saudi guideline do not recommend one option against the other. Zoledronic acid is available in Iran in generic and branded forms. 

## Conclusion

Considering the recommendations of various treatment guidelines, it is obvious that some critical treatment options including enzalutamide, abiraterone, cabazitaxel, and Radium-223 which are recommended in all treatment guidelines should be available and accessible in Iran with average level of health-resources. However, there is also a need for economic evaluations in local setting which allows for selecting the cost-effective options, finding the value-based price, and rational allocation of resources. This recommendation is due to the ration of priority setting in health care and the need for equitable access of the majority of patients in all disease categories to their appropriate treatments.

The economic evaluation and budget impact analyses of health technologies and especially pharmaceuticals are currently performed before registration of new entities in Iran. These evaluations have been mandatory for registering the new molecules in Iran formulary list since 2014. 

The Clinical and pharmacoeconomic assessments are the first steps before registration and market authorization and launching in the pharmaceutical market of Iran. During this process, all medicines are evaluated according to their clinical efficacy and economical effectiveness based on scientific evidences and by scientific committees. After these approvals, the pharmaceutical products have to be registered through issuing CTD (Common Technical Document) to the IFDA (Iran Food and Drug Administration). Consequently, in the pharmaceutical registration process, all technical aspects as well as quality, safety, efficacy, and price are evaluated by expert committees of IFDA.

## Conflict of Interest

The authors declared no conflict of interest
